# On the Application of Gases as a Means of Destroying Contagion

**Published:** 1872-09

**Authors:** 

**Affiliations:** Analyst to the City of Dublin


*On the Application of Gases as a Means of Destroying Contagion.*—A paper read before the Medical Society of the College of Physicians, by Dr. CAMERON, Analyst to the City of Dublin. With discussions.

There is no evidence of a satisfactory nature to prove that the ordinary emanations from decomposing animal or vegetable substances are the cause, *per se*, of any contagious disease.

It is more reasonable to believe that zymotic diseases are each of them produced by the introduction of a specific *virus* or *germ* into the animal economy. It is probable that some zymotics are caused by germs which are incapable of multiplication in the body. Such diseases are not, therefore, contagious in the ordinary sense of that word—that is, they are not propagated by matters which are cast forth from the bodies of the sick. In the valuable Report on Yellow Fever, prepared by Dr. J. C. Nott, and published in the Annual Report of the Board of Health of the City of New York, for 1870-71, very strong evidence is adduced to prove that yellow fever is caused by germs, which are not bred within the body. It is, however, shown that those germs may be transported from place to place in the clothes and baggage of men. Dr. Nott brings forward the most convincing proof that decomposing organic matter does not, *per se*, produce yellow fever; and shows that the germs which cause the malady are devitalized by exposure to a temperature of 32° Fahrenheit.

The use of disinfectants is, however, to be commended, because they preserve the atmosphere free from malodorous gases and vapors. Some kinds of so-called disinfectants are also of great utility as a means of preventing the putrefactive decomposition of organic substances. These disinfectants are properly termed antiseptics. They do not altogether prevent animal and vegetable matters from decay; but they greatly retard that process, and then decomposition without sensible putrescence only takes place.

What is it that we try to destroy when we generate chlorine gas in a room which has been tenanted by a small-pox patient? Is it a gas, or a vapor, or an abnormal condition of one or more of the ordinary constituents of the atmosphere? If the cause of the disease lies in an abnormal condition of the atmosphere—in the occurrence of a "pandemic wave" in that fluid, the disinfection of the air of a particular room would be useless, because, where ordinary ventilation is adopted, the purely gaseous contents of an apartment are wholly renewed many times in an hour. What, therefore, would be the use of disinfecting a room if the atmosphere, on entering it, be already tainted? If cholera, small-pox, rinderpest, and other zymotic and epizootic diseases, are caused by abnormal atmospheric conditions, why is it that they speed along the highways of commerce, that they spread most rapidly as the density of population increases, and that they prevail most in those places where least attention is paid to the removal of organic filth? If the amount of carbonic acid in the atmosphere were increased from its normal proportion of 4 parts in 10,000 parts of atmospheric air to 4 parts in 100, serious disease would be the result; but it would afflict all classes alike, and would ravage the country regions equally with the urban districts.

A careful examination of acknowledged facts relative to nearly all the more important epidemic diseases, fully justifies the belief that each is produced by the intoduction of a *materies morbi*, or germ, or virus, or some palpable substance from the bodies of the sick into those of the healthy; and by that way alone. This view of the mode of propagation of zymotic diseases is, perhaps, most conclusively proved by admitted facts in relation to two contagious diseases—namely, *scabies*, or common itch, and syphilis.

The itch is a good example for the purpose of illustrating the nature of contagion. The *materies morbi* is easily seen; it is an entity, it possesses reproductive powers, begetting its own kind, and it is never found except in the bodies of higher animals. The non-contagionists must admit that, at least in the case of this disease, the theory of the contagionists is proved to demonstration, and simply because the virus of the disease is so large as to be almost seen by the unassisted eye.

In general the contagious matter appears to be excessively minute. Chauveau (*Comptes Rendus*, October 19th, 1868), diluted the liquid taken from the pustules of sheep-pox with 10,000 parts of water, and found that it still retained its power of producing small-pox in the sheep. Vaccine matter from man may be diluted with ten times its weight of water without losing its contagious property to a sensible extent, but if diluted with 500 parts, it becomes perfectly inactive. Hence it is evident that the contagious liquid of sheep-pox is many times more powerful than vaccine, probably because it contains a larger number of the actual particles, or germs that produce disease. These germs have been carefully sought for by eminent pathologists and microscopists. On the whole, the results of the investigations of these inquirers have not been barren. It is shown that vaccine contains, in suspension, minute quantities of two kinds of solid particles—*leucocytes* (which resemble pus corpuscles), and smaller particles not exceeding the 1-20,000th of an inch in diameter. The leucocytes may be easily separated from the other particles and the serum; and they are found to be perfectly inactive. The vaccine property must, therefore, reside either in the small particles or the clear serum. By means of the diffusion apparatus, Burdon-Sanderson and Chauveau have succeeded in obtaining the serum free from the small particles, but failed to produce vaccinia with it either in man or in the ox. These important and accurately conducted experiments prove that the actual cause of cow-pox, and inferentially of other kinds of small-pox, is a minute solid and insoluble body.

It has been strongly urged as an argument against the germ theory of disease that it fails to account for epidemics. Why should small-pox die out in Ireland, and then suddenly reappear and rage with great violence in many parts during the last twelve months? How is it that cholera periodically invades the West from the East? Why does an epidemic gradually increase in intensity, attain a maximum of virulence, and then gradually die out? It is difficult to answer these questions satisfactorily, because all the factors concerned in the propagation of zymotic diseases are not known.

If it be admitted that small-pox and certain other diseases are sometimes caused by matters thrown off from the sick making an entry into the bodies of healthy persons, then the phenomena of epidemics may be shown to be explicable without abandoning the theory that small-pox (and some other diseases) are only communicable from individual to individual. We can readily understand that the low forms of life which produce epidemic and epizootic disease might, under favorable circumstances, multiply to a greater extent than usual. Under such circumstances the chances of their getting into the bodies of animals would be proportionately increased, and a local epidemic would be the result. Intercommunication between the place where the germs were first developed and other places would soon scatter them over areas more or less considerable.

In some epiphytic diseases we find the analogues of epidemic and epizootic maladies. The "blights" in the cereals and other plants are caused by the ravages of minute parasitical fungi. A common disease of wheat grain is occasioned by the presence of the fungus *Uredo caries*, the seeds, or sporules of which are so minute that, according to Bauer, a single grain of wheat may contain 4,000,000 of them. The fungi which produce the diseases of plants do not originate sporadically, nor are they ever found except as parasites. For years a whole locality may be absolutely or comparatively free from them, when suddenly these pests will appear and destroy whole crops. It is the same with respect to the ravages of plants by insects; suddenly the caterpillars of moths will appear in vast numbers in localities where they had previously been very scarce. A few years ago the extensive plantations at Dunsany Castle, county of Meath, became suddenly the abode of myriads of caterpillars, which speedily stripped the bark and leaves of a large proportion of the trees.

If species of bacteria or similar objects are the contagia of certain diseases, then we can understand why it is that so many persons who are near small-pox and fever patients escape, whilst persons not in contact with the infected catch the disease. The bacteria thrown off from the bodies of the sick are not equally diffused throughout the air as a gas or vapor would be, but, for the most part, are scattered about on the clothes and on other solid surfaces, from which they may be conveyed to great distances without making their entry into the body of any one. Contagion in general is conveyed by means of clothes or other solid substances, and is rarely directly propagated through the air. In the Report on Yellow Fever, by J. C. Nott, that writer says:

"No evidence, I think, could be more complete to establish the probability of a disease. All facts being opposed to its contagiousness, I can come to but one conclusion, viz., that the germ may be closed up in trunks or boxes, or be shut up in the baggage car of a railroad, transported from one point to another. I have never seen anybody communicate the disease where luggage was not taken with the patient, and as we find that the disease generally goes everywhere that steamboats go from our infected ports in epidemic years, I see no other conclusion than the one I have before given, viz., that the germ is carried closed up with baggage, and not generated and communicated by personal contagion."

Before the question of bacteria as a cause of zymotics arose, Haygarth, Murchison, Ryan of Lyons, and others, denied that small-pox poison was directly transmitted through any considerable space in the open air. Murchison asserts that the poison was not contagious in the open air at a distance of half a yard. Chauveau states that the contagious matter of small-pox is volatile—that the solid particles float into the air at a temperature of 40 degrees centigrade, but in his experiments the matter was carried away by a current of vapor.

The results of several experiments made by the author show that bacteria and the contagious particles of vaccine lymph resist, when protected by an extremely thin film of solid or semi-solid matters, the action of chlorine and sulphurous acid gases applied to them in larger quantities than are usually employed in disinfection. The filtered meat-juice used in these experiments contained only five grains of solid matter per ounce of 480 grains—less than one per cent. The object glasses were dipped in this liquid, and many of them allowed to drain before being subjected to experiment. We may readily conceive then how extremely thin the film was that separated the bacteria from the gases set free. It is extremely improbable that the actual contagious particles of small-pox or cholera, or similar diseases, are ever detached from the serum and other matter with which they associated when thrown off from the body. They are, no doubt, invested with some such film as that which protects the contagious granules in vaccine. If ordinary gaseous disinfection sometimes fails to destroy the vitality of vaccine, and has no effect on ordinary microsymes, we cannot rely upon it as a means of destroying the contagiums of zymotic diseases which certainly are near akin, if not to bacteria, at least to the virus of vaccine. The recent experiments of Grace Calvert show that bacteria sustain a very high temperature without being killed; and, on the other hand, Melsens, in the *Journal de Pharmacie et de Chimie* for September, 1870, shows that vaccine lymph retains its activity when exposed to the intense cold of 80° centigrade. The low forms of life are often capable of resisting influences which, in the case of the most highly organized animals, would produce fatal results.

No doubt, chlorine, sulphurous acid, and some others of the so-called disinfectants, destroy bacteria and contagia; but in order to do this they must be employed in much larger quantities than they have hitherto been used.

The complete disinfection of a room tainted with the poison of contagious disease can only be accomplished by the most thorough cleansing. The paper should be removed from the walls, and the latter scraped. The ceiling should be washed and whitewashed, the woodwork and floors should be scoured; all these detergent processes remove—probably without destroying them—the contagious particles. The old-fashioned plan of simply whitewashing the walls and ceiling of a room, and washing the woodwork has much to commend it, and it is infinitely more efficacious than gaseous disinfection without liquid applications. If the whitewash does not kill the bacteria, it certainly imprisons them securely.

---

After some remarks from Dr. Malachi Burk, Dr. Grimshaw said that the process of disinfection was carried out by the officers of the Public Health Committee after this fashion. A man comes with a pint of chloride of lime in an old battered tin; he dilutes it with sulphuric acid, places it in a vessel in the middle of the room, shuts it up and leaves it there until the next morning. The usual course when he turned his back was for the owner of the room to throw the disinfectant out of the window. There were but two men at present employed by the Public Health Committee disinfecting the whole of Dublin, so that chemical disinfection, or disinfection by gas, was not fairly tested in this city; and it appeared from a statement in the public journals, that only one in 35 of all the houses reported as being infected by small-pox had been disinfected. On the whole, so far as they knew at present, the only effective disinfectant was a high heat, and even more important were detergent measures. It was only recently it had been announced in Dublin that gas was a disinfectant. He found, on looking over the records of Cork Street Hospital, that formerly such a thing was never attempted as trying to purify a house by fumigation.

Dr. Darby said they did not know what was the cause of epidemic disease. Was it those little animals referred to? It was not at all proved they were the cause of disease, and he did not believe they were. He had come to the conclusion that there was scarcely any disease which affected the human body that might not at one time or other become epidemic. He had been for over 30 years connected with a large public institution, and he had not the slightest doubt, from his experience there, that vermin were sometimes epidemic; that itch was epidemic; that chronic purpura was epidemic. He had had thirty cases of chronic purpura in the hospital at one time after the famine. It was supposed by some that disease was propagated by a germ, but he did not find any satisfactory proof of that proposition. Cerebro-spinal arachnitis was not known in Ireland until it appeared in his hospital. It remained for the late Dr. Mayne to throw more light on the disease than he could. All he could do was to bring case after case forward at the Surgical Society in order to elicit information, but not a man in the room knew anything about it, and many of them thought he had found out a mare's nest. It appeared among healthy boys in the workhouse school, and it attacked none but boys of from 10 to 15 years of age. It was next seen in Belfast, and then it came to Dublin. Take the present epidemic of small-pox. It went about in the same way.

Dr. Burke rose to order, and submitted that Dr. Darby was not speaking to the question.

Dr. Darby believed that plenty of soap and water and pure air were the best disinfectants. When small-pox appeared in the hospital under his care, he placed over the doors of the ward a curtain steeped in a solution of chlorate of lime. He did not know that it did any harm, and he doubted whether it did any good; but he had great faith in fresh air and in cleanliness. The fact that these epidemics come suddenly, rise gradually to a maximum, and then decline, was an argument against the germ theory.

Dr. Finny thought the last two speakers seemed to have misunderstood what Dr. Cameron meant to suggest. Dr. Cameron did not state that bacteria and fever germs were identical, but he assumed that the fever germs were animal or vegetable substances, certain small molecules, each propagating its own specific disease, and no other—the virus of small-pox giving small-pox, that of scarlatina scarlet fever, and that of vaccine cow-pock. He, secondly, assumed that the bacteria, or vibriones, or microzymes, are found in putrescent matters, and also wherever there is any disease that appears to be spreading from one individual to another. He did not, however, assume that the bacteria were identical with the germs of the disease.

Dr. Lyons rose to say that he could not be taken as concurring in the idea that minute microscopic animalculæ or vegetation were the germs or origin of any but a few and well defined forms of cutaneous disease. He thought they were traveling in an entirely wrong direction in looking for the origin of epidemic disease in that quarter. He had himself an idea that diseased processes, however variable in their superficial manifestations, were, when viewed from a profound pathological point of view, more nearly allied, and much more closely similar in their essential conditions than would be supposed from a superficial view of them. He (Dr. Lyons) for one was not prepared to admit that all diseases were propagated by contagion, that like generated like in disease, or that contagion would explain the development of all epidemic diseases. He doubted if, in the present condition of knowledge, any two persons in that room would agree as to what was, and what was not, contagion, or what was to be accepted as the element of contagion in any given case, or, if admitted, how its action was to be explained.

After some observations by Dr. Arthur Wynne Foot, the Chairman thought Dr. Cameron had, to a great extent, established the object he had in view with respect to the action of disinfectants. He had shown that it required a much higher degree of concentration to destroy animalcules than had been heretofore considered necessary. In the course of his observations he alluded to two diseases with which they were familiar, itch and syphilis, as unmistakable examples of contagion. That every one would admit; but they would also admit that there was a wide difference between contagion and epidemic influences. That was an important matter to keep in view. With respect to disinfectants, they all knew how often disinfectants and deodorizers were confounded. As to the germ theory, he was not very well versed in it, but he was not aware of any fact proved that pointed to the existence of particular germs for disease. He thought until they had something more tangible than they had as yet, they were not able to say that disease was propagated by germs. The Metropolitan Sanitary Board of London requested returns to be made from all the vestries who employed people in cleaning the sewers, of the number of persons employed in that way, the age of those persons, the duration of their employment, and the cases of fever amongst them. Two hundred and thirty-four individuals were included in the returns. A great many of them had been engaged seventeen years in that occupation, and the whole sum of the cases of fever amongst them was six. That struck him as a very remarkable event.

Dr. Cameron, in reply, said that Dr. Finny had so clearly put his views before the meeting that he found it unnecessary to recapitulate them. All he could find in Dr. Grimshaw's remarks was that there was a something in contagion which heat destroyed. If that something were any of those abnormal conditions of the air, or loss of nervous energy, of which they had heard, he did not see how an increase of temperature or whitewashing could destroy it. There was a something which was capable of communicating disease from one individual to another; for there could be no doubt there was contagious matter in small-pox, pustules in farcy in the horse, in pleuro-pneumonia in the ox, and in vaccine, and we could produce any of those diseases by introducing a certain kind of morbid matter into the blood of an animal. He did not mean to say chlorine would not do some service. All he wanted to say was, that unless used in very large quantities it did not destroy the lowest forms of life, and then he asked why did they use chlorine at all? The germs of disease, or, at all events, bacteria, did not float in the room, and they were not destroyed by the quantities of the disinfectants ordinarily used. To destroy them larger quantities must be employed, and they should be, in great part, used in solution. He had dipped a brush in a strong solution of chloride of lime, and passed it over a glass on which there were bacteria. He then passed over this, water which had been previously heated to a high degree, and he found the bacteria were destroyed. With regard to syphilis being an epidemic, the first time it was heard of it assumed something of that character.

The only reason why syphilis did not become an epidemic was that its poison was not disseminated through the air as easily as other kinds,—it was more ponderous. Dr. Russell, of Glasgow, a very accurate observer, showed that all the persons connected with the Fever Hospital of that city, the ward-maids, the nurses, the store-keeper, the man at the gate, sooner or later caught fever. There were different degrees of rapidity with which the poison of contagion was propagated. Actual contact was required to give syphilis, whilst small-pox, being more volatile, might be carried in minute particles in the clothes. As to what Dr. Smith had said about sewers, it establishes the theory of contagion. There was no other theory that afforded a satisfactory solution to immunity from epidemic diseases in certain cases. The immunity of the persons employed in cleaning the sewers merely showed that decomposing ordinary animal and vegetable matter, *per se*, would not produce zymotic disease. He knew a family in the country who persistently drank water that contained 20 grains of organic matter per gallon; it had even a bad odor, and it came from a well so situated that the drainage from the stable-yard and out-offices flowed into its shaft. The family, as he had said, drank it continuously, and yet no contagious disease had ever broken out amongst them. Why? The water was impure, it had a bad smell, but the germs of disease were not there. Time was a great factor in disinfection. He found that the quantity of disinfectant matter applied in gaseous form which would not kill bacteria in a short time, would do so after a prolonged contact. They could not properly disinfect a house in less than twenty-four hours. No house in which a small-pox patient had died could be considered free from contagion until the walls were scraped and whitewashed, and the place thoroughly swept out.—*Med. Press and Circular.*

